# Long‐range projections from sparse populations of GABAergic neurons in murine subplate

**DOI:** 10.1002/cne.24592

**Published:** 2019-01-02

**Authors:** Jacqueline Boon, Emma Clarke, Nicoletta Kessaris, André Goffinet, Zoltán Molnár, Anna Hoerder‐Suabedissen

**Affiliations:** ^1^ Department of Physiology, Anatomy and Genetics University of Oxford Oxford United Kingdom; ^2^ Hotchkiss Brain Institute University of Calgary Calgary Alberta Canada; ^3^ Royal Free London NHS Foundation Trust London United Kingdom; ^4^ Wolfson Institute for Biomedical Research and Department of Cell and Developmental Biology University College London London United Kingdom; ^5^ Institute of Neuroscience Université Catholique de Louvain Louvain‐la‐Neuve Belgium

**Keywords:** connective tissue growth factor, GABAergic neuron, RRID: AB_10000240, RRID: AB_10000340, RRID: AB_2298772, RRID: AB_2619710, RRID: AB_477652, RRID: AB_638805, subplate

## Abstract

The murine subplate contains some of the earliest generated populations of neurons in the cerebral cortex, which play an important role in the maturation of cortical inhibition. Here we present multiple lines of evidence, that the subplate itself is only very sparsely populated with GABAergic neurons at postnatal day (P)8. We used three different transgenic mouse lines, each of which labels a subset of GABAergic, ganglionic eminence derived neurons. Dlx5/6‐eGFP labels the most neurons in cortex (on average 11% of NEUN+ cells across all layers at P8) whereas CGE‐derived Lhx6‐Cre::Dlx1‐Venus^fl^ cells are the sparsest (2% of NEUN+ cells across all layers at P8). There is significant variability in the layer distribution of labeled interneurons, with Dlx5/6‐eGFP and Lhx6‐Cre::R26R‐YFP being expressed most abundantly in Layer 5, whereas CGE‐derived Lhx6‐Cre::Dlx1‐Venus^fl^ cells are least abundant in that layer. All three lines label at most 3% of NEUN+ neurons in the subplate, in contrast to L5, in which up to 30% of neurons are GFP+ in Dlx5/6‐eGFP. We assessed all three GABAergic populations for expression of the subplate neuron marker connective tissue growth factor (CTGF). CTGF labels up to two‐thirds of NEUN+ cells in the subplate, but was never found to colocalize with labeled GABAergic neurons in any of the three transgenic strains. Despite the GABAergic neuronal population in the subplate being sparse, long‐distance axonal connection tracing with carbocyanine dyes revealed that some Gad65‐GFP+ subplate cells form long‐range axonal projections to the internal capsule or callosum.

## 1 INTRODUCTION

Subplate neurons, located between the white matter and layer 6a, are amongst the earliest born neurons of the mouse cortex (Angevine & Sidman, 1961; Bystron, Blakemore, & Rakic, 2008; Hoerder‐Suabedissen & Molnár, 2013; Price, Aslam, Tasker, & Gillies, 1997; Smart, Dehay, Giroud, Berland, & Kennedy, 2002). Whereas many subplate neurons are generated in the ventricular zone of the cortical neuroepithelium, the subplate compartment also contains cells derived from the rostral medial telencephalic wall (RMTW; García‐Moreno, López‐Mascaraque, & De Carlos, 2008; Pedraza, Hoerder‐Suabedissen, Albert‐Maestro, Molnár, & De Carlos, 2014)—to date the only brain structure that has been shown to give rise to both interneurons and projection neurons in rodents. The RMTW region gives rise to particularly early born neurons (neurogenesis is finished by E12.5 in this region) that migrate tangentially into the upper part of the cortical neuroepithelium, where they differentiate into projection neurons eventually located within the subplate and GABAergic neurons in the infragranular layers (but excluding the subplate).

Subplate neurons fulfill important roles during development and cortical maturation (Kanold & Luhmann, 2010). They are amongst the first neurons to develop projections (DeCarlos & O'Leary, 1992; Molnár, Adams, & Blakemore, 1998), and they are necessary for thalamocortical axon guidance during embryonic development (Ghosh, Antonini, McConnell, & Shatz, 1990; Grant, Hoerder‐Suabedissen, & Molnár, 2012; Magnani, Hasenpusch‐Theil, & Theil, 2013; McConnell, Ghosh, & Shatz, 1989; Molnár & Blakemore, 1995). The maturation of inhibitory cortical circuitry in Layer 4 requires an intact subplate layer and its constituent cells during the early postnatal period in cats (Kanold, Kara, Reid, & Shatz, 2003; Kanold & Shatz, 2006), and the characteristic barrel cortex cytoarchitecture in rats fails to emerge if the subplate is ablated at birth (Tolner, Sheikh, Yukin, Kaila, & Kanold, 2012). Additionally, subplate neurons are involved in generating cortical oscillations (Dupont, Hanganu, Kilb, Hirsch, & Luhmann, 2006; Yang, Hanganu‐Opatz, Sun, & Luhmann, 2009).

Electrophysiologically, neonatal rat subplate neurons are relatively mature and capable of firing repetitive, nonadapting, fast action potentials (Luhmann, Reiprich, Hanganu, & Kilb, 2000). They receive functional synaptic inputs mediated via AMPA, NMDA, GABA‐A, and glycine receptors (Hanganu, 2001; Kilb et al., 2008). Additionally, stimulation in the subplate layer with an extracellular electrode or the focal application of GABA within the subplate layer gives rise to GABA‐mediated currents in Cajal–Retzius cells of the marginal zone with a time‐course suggestive of monosynaptic connections (Myakhar, Unichenko, & Kirischuk, 2011). Together, this can be taken as evidence of mature GABAergic neurons located within the young postnatal rodent subplate.

In addition to their electrophysiological characterization, subplate neurons have been identified by location within the cortical plate, by their early birth date and their enriched or selective gene expression (Allendoerfer & Shatz, 1994; Kanold & Luhmann, 2010). For embryonic mouse brains, there are at least nine genes with subplate restricted cortical expression and expression in the “superplate” in Reeler brains (Oeschger et al., 2012). Of these, RCAN2 protein colocalizes with Gad67‐GFP in the embryonic (E17.5) and postnatal (P8) subplate (Oeschger et al., 2012), but it only labels a small proportion of all cells in the subplate. For postnatal mouse brains, there are at least an additional five molecules identified that label cells in the subplate region in normal brains, and cells in the superplate in Reeler brains. These include CTGF, NURR1, CPLX3, *Tmem193*, and *MoxD1* (Hoerder‐Suabedissen et al., 2009). Of these, CTGF, CPLX3, and NURR1 have been shown to be expressed in overlapping cell groups, some of which are generated on or before E12.5 (Hoerder‐Suabedissen & Molnár, 2013), that is so called “early born” neurons of the cerebral cortex. Lpar1‐eGFP, expressed from a bacterial artificial chromosome inserted into the Y‐chromosome, is present in subplate cells embryonically, but GFP expression additionally emerges in cortical interneurons in deep layers in the first postnatal week (Hoerder‐Suabedissen & Molnár, 2013; Marques‐Smith et al., 2016). Within the subplate, Lpar1‐eGFP colocalizes at the cellular level with CPLX3, NURR1, and CTGF (Hoerder‐Suabedissen & Molnár, 2013). CPLX3, NURR1, or Lpar1‐eGFP are not expressed in GABA‐expressing or Gad65‐GFP positive interneurons within the subplate region (Hoerder‐Suabedissen et al., 2009; Hoerder‐Suabedissen & Molnár, 2013), nor does Lpar1‐eGFP colocalize with somatostatin in the subplate (Marques‐Smith et al., 2016). In contrast, in humans, the remnants of the subplate, called interstitial white matter neurons, are largely NADPH diaphorase positive interneurons (Akbarian et al., 1996). These NADPH diaphorase positive cells develop by 15 GW in human frontal cortex, in the emergent subplate (Yan, Garey, & Jen, 1996). NADPH diaphorase positive cells in cat white matter and layer 6b can have long‐range projections (Higo, Udaka, & Tamamaki, 2007), and long‐range projecting, nNOS+ cells were also found in the white matter of monkeys (Swiegers et al., 2018; Tomioka & Rockland, 2007).

Here we show, that the most abundant postnatally expressed subplate marker—connective tissue growth factor (CTGF, also known as CCN2), is not present in GABAergic interneurons. CTGF is therefore presumed to be expressed in glutamatergic projection neurons. We quantified the distribution of labeled caudal ganglionic eminence (CGE)‐derived Lhx6‐Cre::Dlx1‐Venus^fl^ GABAergic neurons, medial ganglionic eminence (MGE)‐derived Lhx6‐Cre::R262R‐YFP GABAergic neurons and lateral ganglionic eminence (LGE)/MGE‐derived Dlx5/6‐IRES‐eGFP GABAergic neurons in the mouse primary somatosensory cortex at postnatal day (P)8. Surprisingly, we present data demonstrating that GABAergic neurons are less common in subplate than in other cortical layers in the P8 mouse. None‐the‐less, some of the sparse GABAergic neurons in the subplate as labeled by Gad65‐GFP possess long‐range axonal projections.

## 2 METHODS

### 2.1 Animals

All animal experiments were approved by a local ethical review committee and conducted in accordance with personal and project licenses under the U.K. Animals (Scientific Procedures) Act (1986) and European legislation. All tissue used was from postnatal day (P)8 old mice, of unknown sex unless otherwise specified. Four percent paraformaldehyde (PFA) fixed Dlx5/6‐Cre‐IRES‐eGFP (Stenman, Toresson, & Campbell, 2003) brains were obtained from A. Goffinet (Louvain, Belgium), Lhx6‐Cre::Dlx1‐Venus^fl^ (Kessaris & Rubin, 2013), and Lhx6‐Cre::ROSA26‐YFP (Kessaris & Rubin, 2013) brains were obtained from N. Kessaris (London, UK). Wild‐type NIHS female pups were perfused with and postfixed (for 24 hr) in 4% PFA + 0.25% glutaraldehyde in 0.1 M PBS. E19 Tg(Lpar1‐eGFP)GSat193Mmucd (Hoerder‐Suabedissen & Molnár, 2013) heads were fixed by immersion in 4% PFA overnight. For convenience, the following nomenclature will be used throughout the manuscript: Dlx5/6‐Cre‐IRES‐eGFP will be referred to as Dlx5/6‐eGFP, and Tg(Lpar1‐eGFP)GSat193Mmucd will be referred to as Lpar1‐eGFP.

For carbocyanine dye tracing, Gad65‐GFP (López‐Bendito et al., 2004) mouse pups aged P2 and P7 were killed by Schedule 1 cervical dislocation, brains removed from the skull and fresh brains fixed by immersion in 4% PFA for 24 hr or transcardially perfused with 4% PFA and postfixed in 4% PFA for 24 hr.

### 2.2 Immunohistochemistry

Immunohistochemistry was done on sections from 4% PFA‐fixed brains cut coronally at 50 μm on a vibrating microtome (VT1000S, Leica) and stored in 0.1 M PBS with 0.05% sodium azide until use. Fluorescent triple immunohistochemistry was done on three to four free‐floating sections per brain and antibody combination, all in the region of primary somatosensory cortex.

Following washes with 0.1 M PBS, sections were blocked in blocking solution (2% donkey serum (Sigma) and 1% Triton‐X100 (BDH, Poole, UK) for 2 hr at room temperature (RT) before being incubated overnight at 4 °C with the primary antibodies in the blocking solution. The following antibodies were used: anti‐CPLX3 (rabbit, 122–301, Synaptic Systems, 1:1000), anti‐CTGF (RRID: AB_638805; goat, sc‐14,939, Santa Cruz, 1:500), anti‐GFP (RRID: AB_10000240; chicken, GFP‐1020, Aves Labs, 1:10000), anti‐NEUN (RRID: AB_2298772; mouse, MAB377, Chemicon, 1:100–1:1000 dependent upon lot), anti‐GABA (RRID: AB_477652; rabbit, A2052, Sigma, 1:5000), anti‐Calbindin28K (RRID: AB_10000340; rabbit, CB 38, Swant, 1:5000), and anti‐Calretinin (RRID: AB_2619710; rabbit, 7,697, Swant, 1:2500). Following further washes, the sections were incubated with secondary antibody in lower Triton blocking solution (2% serum +0.1% Triton‐X100) for 2 hr at RT. The following antibodies were used: biotinylated donkey anti‐goat (Abcam, ab97113, 1:100), Alexa568‐conjugated donkey anti‐rabbit (Invitrogen, A10042, 1:500) or donkey anti‐mouse (Invitrogen, A10037, 1:500), and Alexa488 conjugated donkey anti‐chicken (Jackson Immuno Research [Newmarket, UK], 703‐546‐155, 1:500) or donkey anti‐mouse (Invitrogen, A21202, 1:500). Biotinylated secondary antibody was further labeled with streptavidin‐cy5 (1:200; Jackson Immuno Research, S7973‐89K) in blocking solution for 2 hr at RT.

CPLX3 antibody validation: anti‐Complexin 3 antibody was first used and described in the context of retinal ribbon synapses (Reim et al., 2005), and has since been validated additionally by Western blotting against CPLX3‐KO retinal tissue (Reim et al., 2009). The same antibody supplied by Kerstin Reim was used to label subplate neurons (Hoerder‐Suabedissen et al., 2009), as it is in this manuscript, and is now commercially available under the code 122–301 from Synaptic Systems as used in this manuscript.

### 2.3 Definition of subplate and other cortical layers

For the purposes of this study, the subplate zone or layer was defined as a 50 μm thick band directly above the white matter as evident from DAPI or NEUN staining (Hoerder‐Suabedissen & Molnár, 2012), and is equivalent to layer 6b. This corresponds well to the region labeled by CCN2/CTGF, which only rarely labels cells in the underlying white matter. To assess the distribution of GFP or Venus labeled neurons across the entire cortical gray matter depth in primary somatosensory cortex (S1), 50 μm thick bands were placed within each cytoarchitecturally defined layer (i.e. Layer 2–6), well away from the layer boundaries. DAPI counterstaining or NEUN immunohistochemistry was used to identify layer boundaries.

### 2.4 Carbocyanine dye tracing

Brains from Gad65‐GFP positive animals aged P2 or P7 were sectioned on a vibrating microtome (VT1000S; Leica, Germany) to 600 μm coronally. Small crystals of the carbocyanine dye DiI (1,1′‐didodecyl‐3,3,3′,3′‐tetra‐methylindocarbocyanine perchlorate; Invitrogen, Paisley, UK) were placed under visual guidance into the upper cortical layers, the internal capsule or the white matter/corpus callosum into the thick slices. Slices were incubated at 37 °C for 5–10 weeks (Molnár, Blakey, Bystron, & Carney, 2006). Following the incubation, the thick slices were re‐embedded in 5% agarose (Bioline, London, UK) and re‐sectioned to 70 μm on a vibrating microtome, counterstained with bisbenzimide (2.5 μg/100 mL, Hoechst33258; Sigma, Gillingham, UK) or DAPI (Invitrogen) and mounted using 0.1 M PB.

### 2.5 Imaging and analysis

For analysis of GFP+ cell distribution or colocalization of CTGF and GFP, image stacks along the white matter‐cortex boundary or through the entire depth of cortex were acquired on a confocal laser scanning microscope (LSM710, Zeiss) in primary sensory cortex. Imaging intensity and filter‐cutoffs were selected to minimize bleed‐through. All images were subsequently imported into Photoshop CS3 and intensity and contrast adjusted.

To determine the proportion of GFP+ interneurons in each layer, NEUN+ cells that were also GFP+ (or Venus+) were identified. Quantification was based on one to three 50 μm thick bands per layer. To determine the proportion of CTGF+ subplate cells that were also interneurons, NEUN+CTGF+ that were also GFP+ (or Venus+) were identified.

For the analysis of carbocyanine‐labeled sections, an epifluorescence microscope (DMR; Leica, Germany) was used. DiI‐labeled cells were identified and analyzed for GFP fluorescence. There was no visible bleed‐through of either GFP onto the cy3 filter used for DiI or of DiI into the cy2 filter used for GFP on this microscope.

Figures for publication were assembled and contrast and intensity adjusted as a whole using Photoshop CS3 and later versions.

## 3 RESULTS

### 3.1 Enrichment of interneuron markers in the subplate—microarray data

The rodent subplate layer contains a diverse population of cells, containing both pallium derived projection neurons (TBR1+, presumed glutamatergic) and ganglionic eminence derived interneurons (GABAergic; Hevner & Zecevic, 2006). Moreover, it is one of several compartments in which ganglionic eminence derived interneurons migrate tangentially during cortical development (Parnavelas, 2000) and recently subplate has been implicated in regulating radial migration (Ohtaka‐Maruyama et al., 2018). We have previously used microarray profiling of the mouse subplate layer at E15, E18 and P8 with the aim of identifying different cell types within the subplate (Hoerder‐Suabedissen et al., 2013). Of the commonly used interneuron molecular markers (see Supporting Information Table S1 for full list), *Gad1* (*Glutamate decarboxylase 1 [brain, 67 kDa]*; encodes Gad67), *Cck* (*Cholecystokinin*) and *Sst* (*Somatostatin*) are expressed at higher levels in the subplate compared to adjacent cortical plate or Layer 6 in embryonic development, but not postnatally, based on the previously published microarray studies in mouse (Hoerder‐Suabedissen et al., 2009, 2013; Oeschger et al., 2012). *Cck* is expressed in a subplate‐enriched pattern from E14 to E18, but more broadly in subplate and L6 by P4. Similarly, *Sst* expression is primarily in the preplate derived structures of the subplate and marginal zone at E15. *Sst* expressing cells are more abundant in the subplate than in other cortical layers at E18, but by P4, *Sst* expressing cells are distributed across all infragranular layers, and SST+ cells are essentially absent from subplate by P8 (Hoerder‐Suabedissen et al., 2013; Marques‐Smith et al., 2016). *Gad1* expressing cells are sparsely present in the anterior subplate at E15 and E18, but present throughout the cortex by P4.

**Figure 1:**
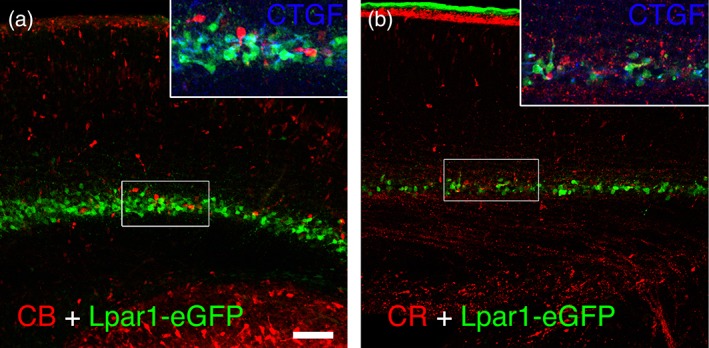
Calbindin but not Calretinin labels the mouse subplate at E19. (a) Calbindin (CB, red) positive cells are present more abundantly in the subplate, labeled here by Lpar1‐eGFP (green), than in cortical plate at E19. CB+ cells are not labeled by CTGF (blue label in inset), nor Lpar1‐eGFP. (b) CR+ cells are rarely present in the subplate at E19, although there are many CR+ fibers extending just below the subplate. Boxed regions in both panels show the region enlarged in the inset. Scale bar = 50 μm (applies to both panels)

The calcium‐binding protein Calretinin, often found in GABAergic cells, has been historically used as a subplate marker during embryonic cortical development, because it labels cells in the preplate derivative structures subplate and marginal zone in the E16 rat cortex (Fonseca, DelRio, Martinez, Gomez, & Soriano, 1995). Calretinin is expressed in the rat subplate from E16 to P3, but expression levels decline sharply after that time point (Fonseca et al., 1995). However, the above gene expression profiling did not identify *Calb2* as differentially expressed between subplate and Layer 6 (or cortical plate) at any of the developmental ages analyzed. In support of this, Figure 1 shows that Calbindin (CB), but not Calretinin cells are relatively more abundant in the E19 mouse subplate than in the cortical plate.

**Table 1:** Summary of brains used for each set of experiments, including age of animal and genetic status

	Carbocyanine tracing		Immunohistochemistry		
	P2	P7	E19	P8	
			CB, CR	CTGF & NEUN	GABA
Gad65‐GFP	9	7			
NIHS					2
Lpar1‐eGFP			2		
Lhx6‐Cre::Dlx1‐Venus^fl^				4	
Lhx6‐Cre::R26R‐YFP				4	
Dlx5/6‐eGFP				5	

On the other hand, most of the molecular markers of subplate neurons identified by the above gene expression profiling approach label non‐GABAergic cells in mouse. Both UNC5C and CDH10 expression is restricted to the embryonic subplate, but neither protein is detectable in GABAergic cells (Oeschger et al., 2012). Similarly, NURR1 and CPLX3 label partially overlapping populations of postnatal subplate neurons in mouse, but neither is detectable in GABAergic cells (Hoerder‐Suabedissen et al., 2009). Furthermore, the CB+ cells located in the E19 subplate are neither CTGF nor Lpar1‐eGFP positive (inset Figure 1; not quantified). Colocalization with CPLX3 was not tested, as this protein only becomes detectable in a few scattered cells in the murine subplate around the time of birth (Hoerder‐Suabedissen et al., 2009).

Other subplate markers label both subplate cells and GABAergic interneurons, but colocalization of these markers with GABA is only rarely observed in the subplate itself. In the transgenic mouse Tg(Lpar1‐eGFP)GX193Gsat/Mmucd, strongly GFP‐labeled cells are present in the embryonic subplate and continue to be present in adult layer 6b. These cells do not express GABA (Hoerder‐Suabedissen & Molnár, 2013). Postnatally, a second population of faintly GFP‐labeled cells emerges in the cortical plate, primarily in infragranular layers, but absent from subplate. Many of these cortical plate cells are GABAergic and some are LHX6+ or SST+ (Hoerder‐Suabedissen & Molnár, 2013; Marques‐Smith et al., 2016). RCAN2+ cells are restricted to the subplate and marginal zone at E17.5 and occasionally colocalize with GABA in the subplate. By P8, a further population of RCAN2+ cells has emerged in the cortical plate, many of which are GABA+, whereas the weakly RCAN2+ cells in the subplate are only occasionally GABAergic (Oeschger et al., 2012).

### 3.2 Cell types labeled in Lhx6‐Cre::R26R‐YFP, Lhx6‐Cre::Dlx1‐Venus^fl^ and Dlx5/6‐GFP

As a means of identifying and labeling selected populations of GABAergic interneurons within the mouse subplate, we used three transgenic lines.

*LIM homeobox 6 (Lhx6)* is expressed in the medial ganglionic eminence. Onset of expression coincides with exit from the cell cycle, and continues to be expressed in Parvalbumin (PV) and Somatostatin (SST) positive GABAergic neurons in the mouse cerebral cortex (Butt et al., 2007; Liodis et al., 2007). Lhx6‐Cre::R26R‐YFP labels all MGE‐derived GABAergic neurons with yellow fluorescent protein (YFP; Fogarty et al., 2007).

*Distal‐less homeobox 1* (*Dlx1*) is expressed in GABAergic neuron progenitors as well as migrating GABAergic neurons (Butt et al., 2007). In the *Dlx1‐Venus* ^*fl*^ transgenic mouse line, the green fluorescent protein Venus is expressed in all cells derived from subpallial progenitor zones including medial, lateral and caudal ganglionic eminence. However, in the presence of CRE‐recombinase, driven by the MGE‐expressed *Lhx6* promoter, Venus is excised, giving a subtractive labeling of cells from the LGE/CGE only (Rubin et al., 2010).

*Dlx5/6*‐*eGFP*, contains the transgene *Cre‐IRES2‐eGFP* under the mouse *distal‐less homeobox5/6* enhancer *id6/id5*. eGFP expression from this enhancer is restricted to the SVZ and mantle zone of the MGE and LGE (Stenman et al., 2003), and labels postmitotic neurons in their final location in cortex, too.

### 3.3 Distribution of interneurons

**Figure 2:**
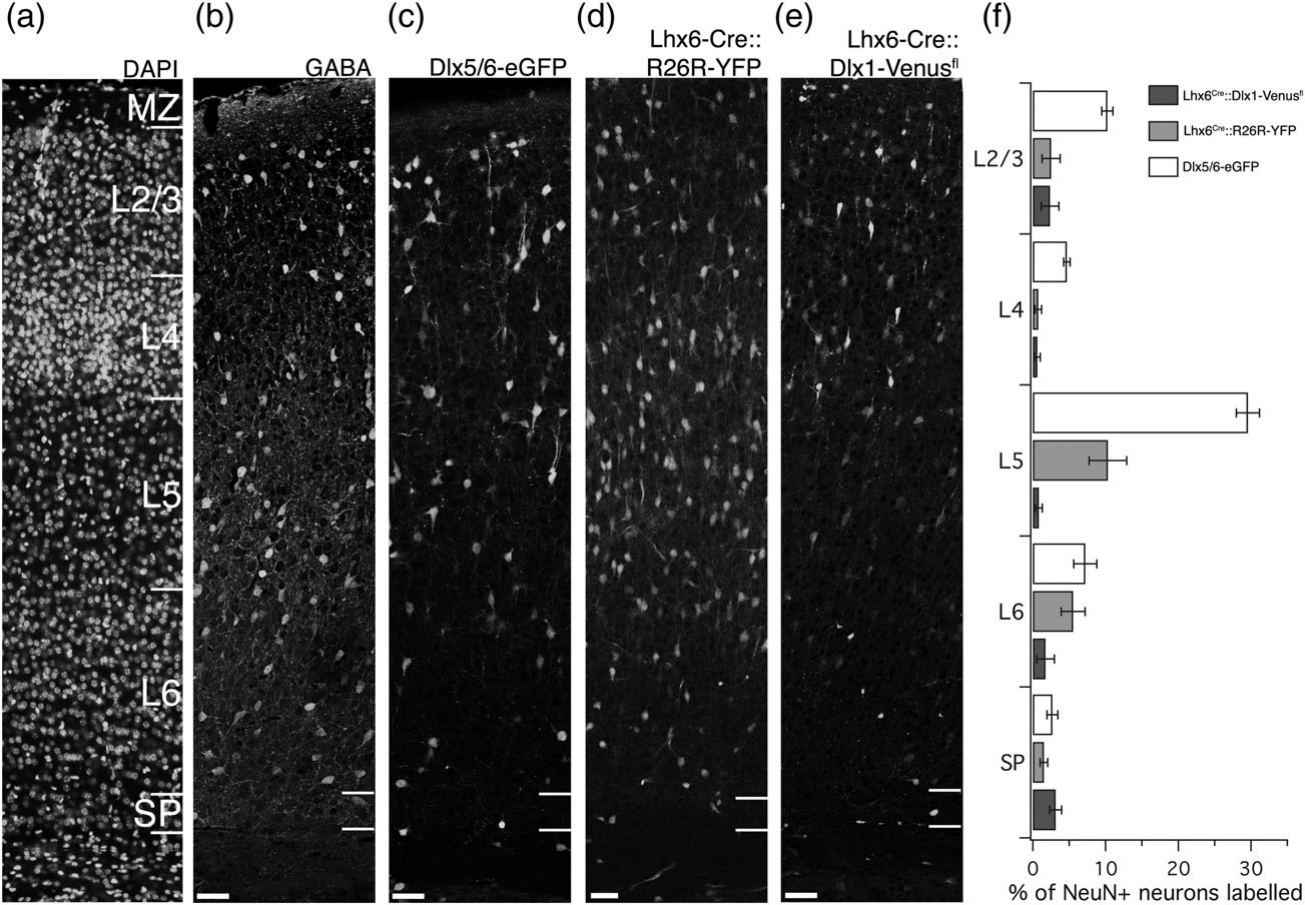
Distribution of labeled GABAergic neurons across all cortical layers at P8. Tiled laser‐scanning confocal microscope images of P8 brains. (a) Example of a DAPI stained section used to delineate layer boundaries in primary somatosensory barrel cortex of all transgenic strains. (b) GABA immunohistochemistry shows abundant GABAergic cells throughout the depth of cortex, with the exception of the subplate, indicated by the white bars near the bottom of the panel. (c) Dlx5/6‐eGFP labels cells interneurons primarily in upper Layer 5 and in Layer 2/3, with hardly any eGFP+ cells located in the subplate. (d) Lhx6‐Cre::R26R‐YFP labels cells throughout most of cortex, but only rarely in subplate. (e) Lhx6‐Cre::Dlx1‐Venus^fl^ labels cells mostly in L2/3 and in subplate, but labels very few GABAergic neurons overall. (f) Labeled GABAergic neurons were quantified as a proportion of all NEUN+ cells identified in one to three 50 μm wide bins within each anatomically defined layer. Data given as mean ± *SEM*. Scale bars = 50 μm

The percentage of interneuron‐marker labeled neurons in primary somatosensory cortex in each of the three transgenic lines analyzed here ranges from 2% (Lhx6‐Cre::Dlx1‐Venus^fl^, *n* = 1,544 NEUN+ cells in cortex in four brains) to 11% (Dlx5/6‐eGFP, *n* = 4,293 NEUN+ cells in cortex in five brains). The NEUN+ interneurons are not uniformly distributed across all cortical layers, and we have quantified the distribution across the cortical layers (Figure 2). For Dlx5/6‐eGFP and Lhx6‐Cre::R26R‐YFP the proportion of labeled cells out of all NEUN+ neurons is greatest in L5, conversely Lhx6‐Cre::Dlx1‐Venus^fl^ is least abundant in L5 and L4. In all three transgenic strains 1–3% of subplate neurons are labeled. Overall, Lhx6‐Cre::Dlx1‐Venus^fl^ labeled the fewest neurons in the primary somatosensory cortex, but had the highest density of labeled neurons in the subplate layer.

### 3.4 Colocalization of CTGF with interneurons in the subplate layer

*Ctgf* is the most abundantly expressed gene in the postnatal subplate (Hoerder‐Suabedissen et al., 2009; Hoerder‐Suabedissen & Molnár, 2013) and adult layer 6b (Belgard et al., 2011; Hoerder‐Suabedissen et al., 2013), both in terms of absolute levels of mRNA produced as well as percentage of neurons labeled. Immunohistochemically, CTGF can be detected in approximately two‐thirds of NEUN+ neurons within the subplate layer in P8 NIHS mouse brains (Hoerder‐Suabedissen & Molnár, 2013), and in 45–65% of subplate neurons in the Lhx6‐Cre::Dlx1‐Venus^fl^, Lhx6‐Cre::R26R‐YFP and Dlx5/6‐eGFP transgenic mouse lines (also at P8). Specifically CTGF labels 47% ± 10 of subplate neurons in Lhx6‐Cre::Dlx1‐Venus^fl^ brains (*n* = 357 cells in four brains), 45% ± 18 of subplate neurons in Lhx6‐Cre::R26R‐YFP brains (*n* = 306 cells in four brains) and 63% ± 9 of subplate neurons in Dlx5/6‐eGFP brains (*n* = 517 cells in 5 brains).

**Figure 3:**
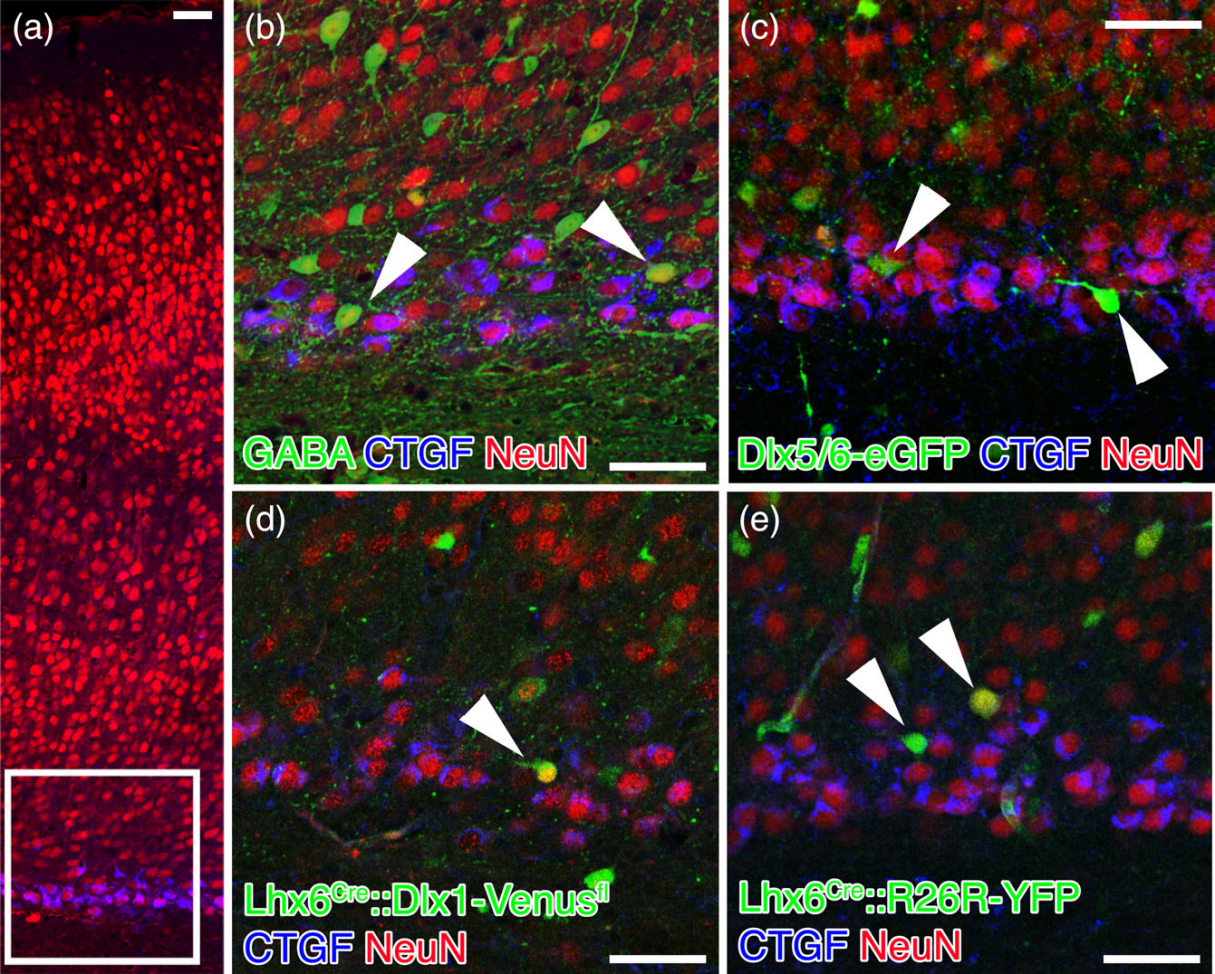
Cells labeled with GABA, Dlx5/6‐eGFP, Lhx6‐Cre::Dlx1‐Venus^fl^ and Lhx6‐Cre::R26R‐eGFP do not colocalize with the subplate marker CTGF in primary somatosensory cortex of P8 mouse brains. (a) Tiled laser‐scanning confocal microscope image of a P8 brain. CTGF+ cells (blue) are only present in the subplate layer, and the white box indicates the approximate area that Panels b–e were imaged at in other sections. (b) GABA+ cells within the subplate are NEUN+ (red), but not CTGF+ (blue). (c–e) some Dlx5/6‐eGFP, Lhx6‐Cre::Dlx1‐Venus^fl^ and Lhx6‐Cre::R26R‐YFP positive cells (arrowheads) within the subplate are NEUN+, but never CTGF+. However, there are also NEUN‐ GABAergic neurons within the subplate, and in particular below the subplate in the white matter (e.g., the green cell in Panel d just above the label). Whereas the NEUN‐ cells within the subplate are usually NEUN+ in another confocal plane, those in the white matter are usually genuinely NEUN‐ at all levels imaged. Scale bars = 50 μm

However, CTGF+ cells do not coexpress GFP/Venus in any of the three transgenic lines used in the present analysis to label GABAergic cells (*n* = 175 CTGF+NEUN+ cells in Lhx6‐Cre::Dlx1‐Venus^fl^, *n* = 131 CTGF+NEUN+ cells in Lhx6‐Cre::R26R‐YFP, and *n* = 325 CTGF+NEUN+ cells in Dlx5/6‐eGFP brains; Figure 3). However, even the most comprehensive labeling of GABAergic neurons seen in the Dlx5/6‐eGFP line still falls considerably short of the general estimate that 20% of cortical neurons are GABAergic. We therefore extended the present study to include triple immunohistochemistry against GABA, CTGF and NEUN.

GABA labeled approximately 6% of NEUN+ neurons in the subplate (59 GABA+NEUN+ cells out of 1,038 NEUN+ cells, *n* = 2 brains). Of these, none were CTGF positive (see Figure 3 for an example).

Neither CPLX3 nor NURR1 colocalize with either GABA or Gad65‐GFP (Hoerder‐Suabedissen et al., 2009) and here we confirmed, that CPLX3 does not colocalize with Dlx5/6‐eGFP either (data not shown, *n* = 196 Cplx3+ cells in 5 brains).

Thus, CTGF is a subplate marker that labels non‐GABAergic, presumed glutamatergic projection neurons in the postnatal subplate. As CTGF protein levels only become detectable in the subplate from E18, it is unlikely to be labeling GABAergic neurons migrating within the subplate at younger ages, but this was not confirmed.

### 3.5 Some of the GABAergic cells in the subplate have long‐range projections

Despite the sparsity of interneurons within the mouse subplate, others have demonstrated that some long‐range, ipsilaterally projecting layer 6b cells are Gad67‐GFP positive in transgenic mice (Tomioka et al., 2005) and long‐range projecting GABAergic neurons have also been described in the cat (Higo et al., 2007) and monkey layer 6b (Tomioka & Rockland, 2007).

Here we assessed whether Gad65‐GFP expressing GABAergic neurons in the subplate and white matter also form long‐range projections to distant targets by carbocyanine dye labeling at P2 and P7. Neurons were labeled by DiI placed in the internal capsule (cells projecting toward the thalamus or other subcortical structures), the distant ipsilateral cortex or the corpus callosum (cells presumed to project contralaterally). A total of *n* = 9 brains at P2 and *n* = 7 brains at P7 were used. Cells located in the subplate or underlying white matter were analyzed for double labeling.

**Figure 4:**
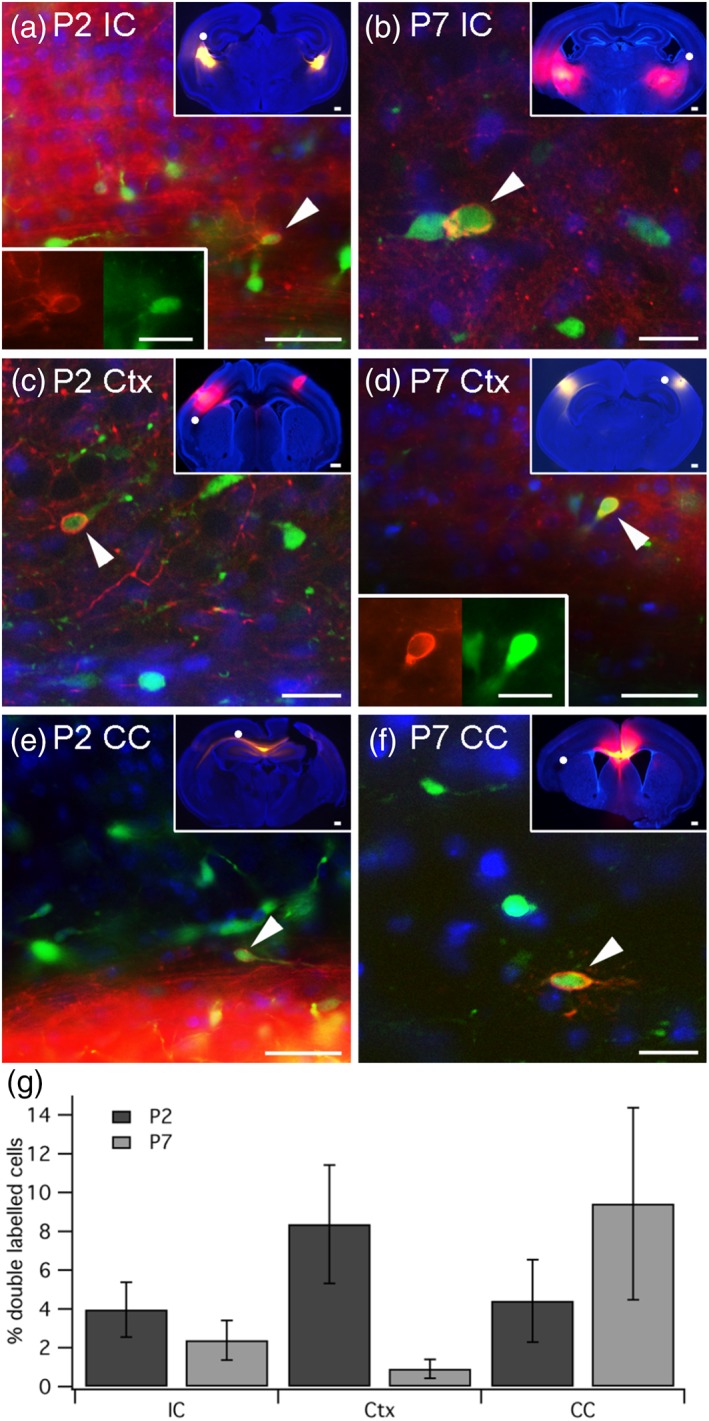
Some Gad65‐GFP+ GABAergic neurons form long‐range projections. (a–f) Epifluorescence microscope images of examples of long‐range projecting subplate cells at P2 or P7 that were traced with DiI (red) from the internal capsule (IC, Panels a + b), ipsilateral but distant (>500 μm) cortex (Ctx, Panels c + d) or corpus callosum (CC, Panels e + f) that are also Gad65‐GFP positive (green) and thus presumed GABAergic neurons. (g) Quantification of the percentage of double DiI + GFP+ cells out of all long‐distance traced cells (mean ± *SEM*). The small insets in Panels a–f show a low magnification image of the slice to indicate the location of the DiI placement. The approximate location of each double labeled cell is indicated with a white dot. The single‐color insets in Panels a and d show the double labeled cell separately in green (GFP) and red (DiI) to make the clear cellular morphology of the DiI signal easier to see. Scale bars = 50 μm, 500 μm, (small insets) and 10 μm (single‐color insets)

DiI labeled cells in the subplate underneath the cingulate, motor, somatosensory, auditory or visual cortex were identified and assessed for double labeling with GFP, thereby counting the percentage of long‐range projecting subplate cells that were Gad65‐GFP+, presumed GABAergic neurons. A total of *n* = 834 and *n* = 1,515 DiI‐labeled cells were assessed for Gad65‐GFP label at P2 and P7 respectively. Gad65‐GFP positive cells were found labeled from all three locations at both ages (see Figure 4 for examples). A dramatic decrease in long‐range projecting GABAergic neurons was identified for cortical long‐distance projecting cells. 8.4% ± 3.1 (*n* = 20/323) of cells traced from distant (>500 μm) cortex at P2 were GABAergic neurons, compared to only 1% ± 0.5 (*n* = 5/532) at P7. It should be noted that the majority of dye placements (7/12 slices) analyzed at P7 did not result in double‐labeled cells, whereas the majority at P2 (6/7 slices) resulted in one or more double labeled cells. Similarly, 4% ± 1.4 of cells (*n* = 15/394) traced from the internal capsule at P2 were GABAergic neurons, compared to 2.4% ± 1.0 (*n* = 16/732) at P7. Conversely, the percentage of GABAergic, subplate cells with an axon in the midline corpus callosum (presumed to project to the contralateral hemisphere) went up from 4.4% ± 2.1 (*n* = 5/117) at P2 to 9.4% ± 5.0 (*n* = 18/251) at P7.

When placing DiI in the internal capsule or the cortex, the smallest possible crystal was used, while relatively large crystals were used for DiI placements into the white matter/ corpus callosum, irrespective of the age or tissue type used.

In conclusion, some long‐range projecting cells of the mouse subplate are Gad65‐GFP+, and therefore likely to be GABAergic neurons, however, they make up less than 5% of the long‐range projecting population of back‐labeled cells. These Gad65‐GFP+ cells project to the internal capsule and thus probably to the thalamus as well as to the corpus callosum and thus probably to the contralateral cortex. Dye was placed in the dense axon bundles of the internal capsule and corpus callosum to increase the likelihood of obtaining back‐labeled cells in the absence of knowledge about the precise target regions of these projections.

## 4 DISCUSSION

Throughout this manuscript, we define the subplate zone or layer as a 50 μm thick band directly above the white matter. Molecular markers CTGF and CPLX3 label cells in approximately this band throughout young postnatal mouse brain development and into adulthood (Hoerder‐Suabedissen et al., 2009; Hoerder‐Suabedissen & Molnár, 2013). CTGF additionally labels some cells within the underlying white matter. In humans and other large‐brained species, it has been proposed that the cells occupying the histologically distinct subplate zone of the embryonic and fetal brain are displaced into the underlying white matter during postnatal development (Judaš, Sedmak, & Pletikos, 2010). While we cannot exclude that some cells initially located within the subplate zone in mice are subsequently displaced into the adult white matter, for the purposes of this study, we have focused on the postnatal subplate zone that remains histologically distinct.

Our study revealed relatively low density of interneurons within the P8 murine subplate. Overall, it is believed that the mouse neocortex consists of approximately 20% interneurons (Gonchar & Burkhalter, 1997), compared to the 25% to 30% found in primate visual cortex (Jones, 1993). Overall, the density of interneurons is higher in Layers 2–5 than in Layer 1 (Gonchar & Burkhalter, 1997; Xu, Roby, & Callaway, 2010). In agreement with this, Tamamaki et al. (2003) reported that 19% of NEUN+ neocortical neurons are GFP+ in the Gad67‐GFP knock‐in mouse line (Tamamaki et al., 2003). However, all of these studies restricted their quantification to layer 6a and the thin, compact layer of 6b has not been specifically investigated. The only evidence for sparse GABAergic neuron presence in the subplate prior to our current study comes from Hevner and Zecevic (2006). They report that subplate “projection” neurons (TBR1+) are born between E10.5 and E13.5 whereas GABAergic cells are born both early and late (E10.5 to E16.5). Assuming that BrdU labeling is distributed evenly across both cell types, summing up all the labeled cells in their Figure suggests that TBR1+ cells outnumber GABA+ cells by at least 10 to 1 at birth (Hevner & Zecevic, 2006), which would be more in agreement with our findings. Here we report that less than 5% of neurons in the mouse subplate are GABAergic in each of three transgenic lines at P8. This is likely to still be an overestimate, as our confocal images were taken of specifically selected areas that included at least one GFP+ cell in the subplate. Similarly, when labeling against GABA, the neurotransmitter released by GABAergic neurons, only 6% of NEUN+ neurons in the subplate are labeled. Lastly, the subplate layer appears unusual in the sense that most labeled GABAergic neurons in the subplate were identified in the Lhx6‐Cre::Dlx1‐Venus^fl^ mouse line, in which overall the fewest GABAergic neurons were labeled.

We quantified only NEUN+ neurons for this analysis, as we noted a relatively large number of GABAergic‐marker positive cells with the distinct morphology of migrating cells at the lower border of the subplate. This suggests that cell migration from the SVZ toward the rostral migratory stream is not entirely restricted to the white matter, and that cells can occasionally enter the overlying subplate. We therefore aimed to exclude migratory GABAergic neurons by colabeling with NEUN. This allowed us to determine the overall distribution of labeled GABAergic neurons out of total neurons as well (see Figure 2).

CTGF only labels a small region of the cytoplasm in most neurons, usually between nucleus and base of the primary dendrite. Therefore, we analyzed colocalization on image stacks to exclude cellular arrangements in which the CTGF is derived from one cell but closely apposed to another cell. However, we only found one example of closely adjacent, but nonoverlapping cells and no genuine colocalization between CTGF and any of the GABAergic markers or transgenic labels. This strongly suggests that CTGF is not coexpressed in GABAergic cells of the mouse subplate.

Even in early brain development, nonmigratory GABAergic neurons may not be abundant in the subplate. Of the many subplate‐selectively expressed genes in embryonic development, only RCAN2 could be demonstrated to colocalize with Gad65‐GFP occasionally (Oeschger et al., 2012). Calretinin, often considered a GABAergic marker, labels GABAergic cells in the E16 rat cortical plate, but non‐GABAergic cells transiently in the subplate (Fonseca et al., 1995).

In light of the unusual mouse subplate composition, it would be interesting to investigate subplate in large‐brained species in more detail (see Swiegers et al., 2018). Previously, it has been noted that NADPH diaphorase labeled cells are largely GABAergic, but specifically within the primate white matter, the majority of NADPHd cells are not GABAergic. Moreover, NADPHd cells within the remaining cortex are often identified as Martinotti cells (Estrada & DeFelipe, 1998; Yan et al., 1996). This in turn, links the interstitial white matter NADPHd cells with Lpar1‐eGFP subplate cells in the mouse, as the latter transgene is also expressed in cortical Martinotti cells (Marques‐Smith et al., 2016). In fact, the Lpar1‐eGFP+ subplate population and the Martinotti cells may be linked by an extracortical site of origin, namely the rostral medial telencephalic wall (Pedraza et al., 2014).

Although they are sparse, GABAergic neurons within the mouse subplate/layer 6b may carry out important circuit functions, as a small proportion forms long‐range projections. GABAergic neurons are also generally referred to as “interneurons”. The small, but significant population of long‐range GABAergic neuron population in the subplate argues against this practice. Here we present evidence that Gad65‐GFP labeled GABAergic neurons can form long processes to subcortical targets (backlabeled with carbocyanine placements in the internal capsule), to the contralateral hemisphere (backlabeled with carbocyanine placements in the corpus callosum) and to distant ipsilateral cortical areas. Similarly, ipsilateral long‐range projections of Gad67‐GFP expressing layer 6b cells had been previously reported for adult mouse brains (Tomioka et al., 2005). We noted a decrease in the percentage of subcortically projecting interneurons during the first postnatal week, but a concomitant increase in contralaterally projecting Gad65‐GFP+ cells. Without additional birthdating (which is incompatible with carbocyanine tracing), it is not possible to determine whether contralaterally projecting GABAergic subplate cells preferentially survive during the first postnatal week, or whether long‐range axons to other targets are withdrawn during this time window. However, we would like to highlight, that other subplate and layer 6b cells also form long‐range projections including to the contralateral hemisphere (Hoerder‐Suabedissen & Molnár, 2012), some of which persist into adulthood (Hoerder‐Suabedissen et al., 2018). It would be interesting to follow this up in mature brains, using alternative methods to carbocyanine dye tracing, which is not a method suitable beyond the end of the second postnatal week.

We only used carbocyanine tracing from a single location per hemisphere or thick section, and therefore cannot comment on whether long‐range projecting GABAergic neurons have multiple distant axonal projections, or project to one location exclusively. This might be interesting to follow up, as subplate neurons have previously been shown to project to distinct distal targets, and in particular, for the innervation of multiple targets to decrease between P2 and P7 (Hoerder‐Suabedissen & Molnár, 2012), which could account for the decrease in the percentage of long‐range projecting GABAergic cells observed here.

Each dye crystal placed in ipsilateral cortex typically covered several cortical layers, and dye for identification of contralateral projections was placed near the corpus callosum, to maximize the chances of finding any long‐range projecting GABAergic cells. This method, unfortunately, precludes detailed analysis of the cortical layer in which long‐range GABAergic cells terminate at distant cortical sites.

In conclusion, we highlight the sparsity of GABAergic neurons within the postnatal mouse subplate layer, and confirm that CTGF, like the other postnatal subplate specific markers CPLX3, NURR1 and Lpar1‐eGFP (Hoerder‐Suabedissen et al., 2009; Hoerder‐Suabedissen & Molnár, 2013) and embryonic subplate specific markers UNC5C and CDH10 (Oeschger et al., 2012), does not label GABAergic neurons. We also confirm that a small proportion of subplate GABAergic neurons forms long range projections to contra‐ and ipsilateral cortex and subcortical targets. It merits further investigation, why the subplate layer specifically appears to have little GABAergic neurons, and what circuit function the long‐range GABAergic projections might carry out. How does the subplate develop differently from the rest of the cortical layers? Or are its transient and dynamic activity patterns of a different nature?

## Supporting information


**Supplementary Table 1** This table lists the commonly used GABAergic neuron molecular markers and whether or not they were identified for subplate enriched gene expression in our previous microarray study comparing subplate gene expression with overlying cortical plate (E15) or layer 6a (E18 and P8; Hoerder‐Suabedissen et al., 2009, 2013; Oeschger et al., 2012). Subplate enriched genes are given anatomical descriptions based on Allen Brain Atlas (Lein et al., 2007) and Genepaint in situ hybridization images as well as the fold‐change enrichment as reported by the microarrays (Hoerder‐Suabedissen et al., 2009, 2013; Oeschger et al., 2012).
