# Supplementary material for: Long‐range projections from sparse populations of GABAergic neurons in murine subplate
Source: J Comp Neurol. 2019 Jan 2;527(10):1610–20. doi: 10.1002/cne.24592 (PMC6492162; doi:10.1002/cne.24592)
Supplement: Supplementary file 1 — Supplementary Table 1 This table lists the commonly used GABAergic neuron molecular markers and whether or not they were identified for subplate enriched gene expression in our previous microarray study comparing subplate gene expression with overlying cortical plate (E15) or layer 6a (E18 and P8; Hoerder‐Suabedissen et al., 2009, 2013; Oeschger et al., 2012). Subplate enriched genes are given anatomical descriptions based on Allen Brain Atlas (Lein et al., 2007) and Genepaint in situ hybridization images as well as the fold‐change enrichment as reported by the microarrays (Hoerder‐Suabedissen et al., 2009, 2013; Oeschger et al., 2012). [file CNE-527-1610-s001.docx]

| Marker | Gene name | Microarray E15 | ISH at E15 | Microarray E18 | ISH at E18 | Microarray at P8 | ISH at P4, P7 or P8 |
| --- | --- | --- | --- | --- | --- | --- | --- |
| Calbindin (CB) | *Calb1* | n/a | -- | n/a | -- | n/a | -- |
| Calretinin (CR) | *Calb2* | n/a | -- | n/a | -- | n/a | -- |
| Colecystokinin (CCK) | *Cck* or *cckn* | 1.5 | SP and lower CP | n/a | SP | n/a | SP and L6 |
| Distal-less homeobox 1 (Dlx1) | *Dlx1* | n/a | Not enriched | 2.6 | Scattered cells in MZ and WM | n/a | Not enriched |
| Distal-less homeobox 2 (Dlx2) | *Dlx2* | n/a | Not enriched | 1.8 | Strong in WM/SP | n/a | Not enriched |
| Distal-less homeobox 5 (Dlx5) | *Dlx5* | n/a | -- | n/a | -- | n/a | -- |
| Distal-less homeobox 6 (Dlx6) | *Dlx6* | n/a | -- | n/a | -- | n/a | -- |
| Glutamate decarboxylase, brain, 65kDa (Gad65) | *Gad2* | n/a | -- | n/a | -- | n/a | -- |
| Glutamate decarboxylase, brain, 67kDa (Gad67) | *Gad1* | n/a | Scattered faint cells in SP | 1.6 | Scattered faint cells in SP | n/a | Not enriched |
| LIM homeobox 6 (Lhx6) | *Lhx6* | n/a | -- | n/a | -- | n/a | -- |
| 5-HT3aR | *Htr3a* | n/a | -- | n/a | -- | n/a | -- |
| Neuropeptide Y (NPY) | *Npy* or *pyy4* | n/a | -- | n/a | -- | n/a | -- |
| NK2 Homebox 2 (Nkx2.2) | *Nkx2-1* | -- | -- | -- | -- | -- | -- |
| Parvalbumin (PV) | *Pvalb* | n/a | -- | n/a | -- | n/a | -- |
| Somatostatin (SS) | *Sst* or *ss1* | 1.7 | Anterior SP and MZ | n/a | Most abundant in SP | n/a | Not enriched |

Supplementary Table 1: GABAergic neuron related gene expression in mouse subplate summarised throughout development. Microarray results are given as significant fold-changes (increase in expression when comparing subplate with cortical plate or L6a) at each age tested (or n/a if the gene was either not present on the microarray, or no significant fold-change was detected), in situ hybridisation results are summarised descriptively. The underlying raw data was published in Hoerder-Suabedissen et al., 2013.
